# Vorsprung durch Technik? Zur Rolle von Technikbereitschaft und Technikausstattung für das Studieren von zuhause

**DOI:** 10.1007/s35834-021-00322-6

**Published:** 2021-09-27

**Authors:** Marios Karapanos, Patrick Hawlitschek

**Affiliations:** 1 Institut für Bildungswissenschaften, Universität Leipzig, Leipzig, Deutschland; 2 Institut zur Qualitätsentwicklung im Bildungswesen (IQB), Humboldt-Universität zu Berlin, Berlin, Deutschland

**Keywords:** Technikbereitschaft, Technikausstattung, Hochschullehre, CoViD-19, Technology commitment, Technical equipment, Higher education, CoViD-19

## Abstract

Seit Beginn der Coronapandemie müssen Studentinnen und Studenten stärker denn je für das Studium auf Technik zurückgreifen. Bislang ist unklar, welche differentiellen Effekte sich durch interindividuelle Unterschiede in der technischen Ausstattung und in der Bereitschaft zum Umgang mit Technik für die Bewältigung von Studienanforderungen und die Zufriedenheit mit den angebotenen Lernmedien ergeben. Die Analyse von Befragungsdaten (*N* = 3332) mit Hilfe von Strukturgleichungsmodellen zeigt, dass beide Ressourcen interindividuelle Unterschiede in der Bewältigung von Lernaktivitäten (Δ*R*^*2*^ = 0,11), der Studienorganisation (Δ*R*^*2*^ = 0,16) und in der Zufriedenheit mit den digitalen Lernmedien (Δ*R*^*2*^ = 0,13) erklären. Technische Ausstattung und Technikbereitschaft erweisen sich dabei für die Bewältigung der untersuchten Studienanforderungen als vergleichbar bedeutsam. Die Zufriedenheit mit den digitalen Lernmedien hingegen scheint stärker an die technische Ausstattung gebunden zu sein. Die Ergebnisse erweitern den derzeit an Hochschulen stattfindenden Diskurs über gute digitale Hochschullehre und verdeutlichen die Notwendigkeit, Lehren und Lernen an Ressourcen der Studentinnen und Studenten auszurichten.

## Abstract (en)

Since the beginning of the Corona pandemic, students have had to rely more than ever on technology to manage their courses. So far, it is unclear what differential effects emerge from interindividual differences in technical equipment, and the willingness to use technology for coping with study requirements, and how both resources are related to student satisfaction with the learning media. Using structural equation models with survey data (*N* = 3332), we show that technical equipment and technology commitment explain interindividual differences in dealing with learning activities (Δ*R*^*2*^ = 0.11), study organization (Δ*R*^*2*^ = 0.16), and in satisfaction with digital learning media (Δ*R*^*2*^ = 0.13). Technical equipment and technology commitment prove to be of comparable relevance for coping with the study requirements investigated. In contrast, satisfaction with digital learning media appears to be linked more strongly to technical equipment. Our results extend the discourse currently taking place in higher education about good teaching and they highlight the need to adapt teaching and learning to student resources.

## Einführung

Seit dem Sommersemester 2020 unterzieht die Coronapandemie Hochschulen weltweit einem enormen Stresstest (Crawford et al. 2020). Für Studentinnen und Studenten bedeutet das in erster Linie, dass Studium und Lehre fast vollumfänglich ohne Präsenz stattfinden mussten und müssen. Obwohl den Hochschulen mit dem Übergang zur technikgestützten Distanzlehre retrospektiv eine „respektable Notlösung“ (Sommer 2020) gelang, sind viele Fragen zur Bewältigung studienrelevanter Anforderungen (z. B. Organisation von Lern- und Studienaktivitäten) und zur Zufriedenheit mit der technikgestützten Hochschullehre noch ungeklärt. Bereits erste Adhoc-Erhebungen unter Studentinnen und Studenten aus dem Frühjahr 2020 deuteten auf große Unsicherheiten hin, auch in technischen Fragen. Zwar ist aus regelmäßig durchgeführten Kinder- und Jugendstudien bekannt, dass Jugendliche und junge Erwachse sehr umfassend mit internetfähigen Endgeräten ausgestattet sind (Feierabend et al. 2019), ein Befund der auch von Untersuchungen zum studienbezogenen Mediennutzungsverhalten gestützt wird (Grosch und Gidion 2011; Karapanos und Fendler 2015; Zawacki-Richter 2015). Schon frühzeitig lagen aber auch Hinweise vor, die zumindest bei einem Teil der Studentinnen und Studenten auf eine mangelhafte technische Ausstattung hindeuteten (Meißelbach und Bochmann 2020; Stammen und Ebert 2020). Da nicht nur Studentinnen und Studenten zum Lernen nach Hause geschickt wurden, sondern auch viele Beschäftigte, Schülerinnen und Schüler von zuhause aus ihren beruflichen bzw. schulischen Verpflichtungen nachgingen, führte das in vielen Ländern zu einem sprunghaften Anstieg bei der Nachfrage nach Kommunikationstechnik (Konishi et al. 2021; Lazarov 2020) bei gleichzeitig sinkendem Angebot durch Schließungen im stationären Handel und den Zusammenbruch von Lieferketten. Eine Preisauswertung des Verbraucherportals Testberichte.de (2021) zeigt, dass insbesondere Webcams schnell vergriffen und nur noch zu Preisen erhältlich waren, die im Mittel fast dem Doppelten des Vorkrisenniveaus entsprachen. Neben technischer Ausstattung erfordert das Studieren von zuhause aber auch eine entsprechende Bereitschaft zum Umgang mit ebendieser Technik. Welche Rolle beide Ressourcen, Technik und technikbezogene Einstellungen und Überzeugungen für die Bewältigung des Studiums und damit den Studienerfolg seit Beginn der Coronapandemie spielen, ist bisher kaum untersucht.

## Theorie und Forschungsstand

### Technikausstattung von Studentinnen und Studenten

Die kommunikationstechnische Ausstattung junger Erwachsener hat sich in den vergangenen Jahren auf hohem Niveau stabilisiert. Nach Ergebnissen der JIM-Studie, die die Situation in Deutschland beschreibt, besitzt in der Altersgruppe der 18- bis 19-Jährigen nahezu jede(r) ein eigenes Smartphone (99 %) und hat über WLAN (97 %) Zugang zum Internet (Feierabend et al. 2019). Etwas mehr als die Hälfte (60 %) besitzt einen eigenen Laptop, Desktop-Computer (39 %) und Tablets (29 %) sind weniger verbreitet (ebenda). Befragungen an deutschen Hochschulen kamen regelmäßig zu vergleichbaren Ergebnissen (Grosch und Gidion 2011; Zawacki-Richter 2015). Bei einer Befragung an der Universität Duisburg-Essen (*N* = 7012) im Sommersemester 2020 gaben allerdings nur drei von vier (75 %) der befragten Studentinnen und Studenten an, alle Geräte zur Verfügung zu haben, die sie für das Onlinesemester benötigten. Mehr noch: Etwas mehr als ein Viertel (28 %) sah eine Neuanschaffung als notwendig an, konnte sich diese aber nicht leisten. Auch bei der Internetverbindung gab es bei einem kleinen Teil der Befragten Defizite. So hatten etwa 8 % lediglich Zugriff über das Mobilfunknetz oder über ein WLAN Dritter, auf dessen Verfügbarkeit sie keinen Einfluss hatten (Stammen und Ebert 2020). Etwas nachteiliger zeichnet eine Befragung (*N* = 3469) im sächsischen Hochschulraum die Situation. Hier gaben nur 72 % der befragten Studentinnen und Studenten an, über eine ausreichend stabile Internetverbindung zu verfügen, die auch für Livestreaming, Videotelefonie und den Download großer Dateien uneingeschränkt geeignet sei (Karapanos et al. 2021). Etwa jede(r) Siebente berichtete von Mängeln bei Peripheriegeräten wie Webcam, Mikrofon oder einem ausreichend großen Bildschirm (ebenda). Dozentinnen und Dozenten wiederum gingen überwiegend davon aus, dass ihre Studentinnen und Studenten über die notwendige technische Ausstattung verfügten. Nur sehr selten fragten sie gezielt nach, um Lehr-Lern-Prozesse auf die technische Ausstattung der Studentinnen und Studenten abzustimmen (ebenda).

Während die Mehrheit der Studentinnen und Studenten zumindest in Deutschland zwar hinsichtlich der technischen Ausstattung gut gerüstet ins digitale Sommersemester 2020 starten konnte, meldete eine nicht zu vernachlässigende Minderheit Defizite an. Unstreitig genügt die reine Verfügbarkeit geeigneter Technik noch nicht für ein erfolgreiches Studieren von zuhause. Es bedarf außerdem der notwendigen Bereitschaft, Technik zielgerichtet zur Bewältigung studiumsbezogener Aufgaben auch einzusetzen.

### Technikbereitschaft

Unter Technikbereitschaft verstehen wir mit Neyer et al. (2012) generalisierte Einstellungen und Überzeugungen einer Person zum Umgang mit Technik. Damit wird ein persönlichkeitspsychologisch erweitertes Modell zur Vorhersage von Techniknutzung zugrunde gelegt, das eine konzeptionelle Weiterentwicklung des Technikakzeptanzmodells (Davis 1989) und der Theorie des überlegten Handelns (Ajzen 1991) darstellt.

Technikbereitschaft umfasst Technikakzeptanz, -kompetenz- und -kontrollüberzeugungen. Technikakzeptanz wird „als ein explizit repräsentiertes Einstellungsmerkmal [definiert], das die subjektive Bewertung technologischen Fortschritts widerspiegelt“ (Neyer et al. 2012, S. 88). Unter Technikkompetenzüberzeugungen sind „subjektive Erwartung von Handlungsmöglichkeiten in technikrelevanten Situationen“ zusammenzufassen, die „als bereichsspezifisches Selbstkonzept eigener Fähigkeiten repräsentiert“ sind (ebenda). Technikkontrollüberzeugungen hingegen bezeichnen „individuelle Kontingenzerwartungen […], welche die subjektive Erwartung der Ergebnisse technikrelevanter Handlungen darstellen“, sie widerspiegeln „das Ausmaß wahrgenommener Kontrollierbarkeit von Technik“ (ebenda). Technikbereitschaft weist damit konzeptuelle Überschneidungen mit anderen technikbezogenen kognitiven (*Computer*/*ICT*^1^* Self Efficacy; *Compeau und Higgins 1995; Hatlevik et al. 2018) und kognitiv-affektiven (*Computer Anxiety*; Chua et al. 1999) Selbstwahrnehmungen auf, legt aber einen breiter gefassten Technikbegriff zugrunde, der nicht nur computertechnische Systeme im engeren Sinne umfasst.

Bisherige Erfahrungen mit Technik wirken sich einerseits auf einer generellen Ebene auf die Entwicklung technikbezogener Kompetenzüberzeugungen aus (Hatlevik et al. 2018), die wiederum entscheidend für die Akzeptanz speziell auch von Lernmedien sind (Cheon et al. 2012; Hsia et al. 2014; Šumak et al. 2011). Bei positiver Ausprägung begünstigen sie andererseits aber auch auf lernspezifischer Ebene bspw. die Lernmotivation (Geng et al. 2019) oder die Wahl effektiver Lernstrategien (Wang et al. 2013). Erkenntnisse zu Effekten technikbezogener Einstellungen und Kompetenzen auf akademisches Lern- und Leistungsverhalten liegen aus internationalen Schulleistungsstudien vor. Untersuchungen auf Basis von PISA-Daten zeigen hierzu gemischte Befunde (Odell et al. 2020). Selbst in Untersuchungen, die auf positive Zusammenhänge hindeuten, fallen diese ausgesprochen klein aus (Hu et al. 2018; Kunina-Habenicht und Goldhammer 2020). Darin widerspiegelt sich vermutlich die geringe Bedeutung, die Informations- und Kommunikationstechnik allgemein für den Schulunterricht – zumindest bis zum Beginn der Coronapandemie – noch besaß. Im rein technikvermittelten Studium jedoch könnte Technikbereitschaft eine prominentere Rolle zur Beschreibung interindividueller Unterschiede bei Lern- und Organisationsprozessen zukommen.

### Lehr-Lern-Prozesse in Hochschulen

Der Terminologie von Angebots-Nutzungs-Modellen folgend bieten Hochschulen ein Lernangebot, das von Studentinnen und Studenten genutzt werden kann (Bernholt et al. 2018). Die Nutzung von Lernangeboten variiert mit individuellen Merkmalen der Lernenden. Da das Lernangebot während der Pandemie vorwiegend aus digitalen Medien besteht, werden die technische Ausstattung und Technikbereitschaft für das Lernen und dessen Organisation relevant, da – so ist zu vermuten – auch diese als individuelle Merkmale die Angebotsnutzung modulieren.

Neben Angebots-Nutzungs-Modellen, die zur heuristischen Beschreibung hochschulischen Lernens bereits in der deutschsprachigen Hochschulforschung herangezogen werden (Bernholt et al. 2018; Braun et al. 2014), betrachtet auch die Studienerfolgsforschung in ähnlicher Weise die Wechselwirkung institutioneller und individueller Aspekte für ‚erfolgreiches‘ Studieren (Bosse und Trautwein 2014; Heublein 2014). Neben dem Erhalt von Bildungszertifikaten dienen häufig Studiennoten, der spätere Berufserfolg oder die selbstberichtete Studienzufriedenheit als empirische Indikatoren von Studienerfolg (Rindermann und Oubaid 1999). Aus interaktionistischer Perspektive spielt die Bewältigung studienspezifischer Anforderungen eine wichtige Rolle zu dessen Bewertung (Bosse und Trautwein 2014; Jänsch und Bosse 2018). Darunter sind bspw. Lernanforderungen (z. B. Lernaktivitäten zeitlich zu strukturieren) oder die Organisation des eigenen Studiums (z. B. den eigenen Stundenplan zu erstellen) zu verstehen.

Hochschulisches Lernen stellt im Vergleich zum schulischen Lernen erweiterte Anforderungen an die selbstgesteuerte Angebotsnutzung. Anders als die Betrachtung von Noten oder Zertifikaten bietet der Fokus auf die Bewältigung von Studienanforderungen eine prozessorientierte Sicht darauf, wie Studentinnen und Studenten innerhalb des gegebenen Rahmens ihrer Hochschule im Studium zurechtkommen. Dies gelingt besser, wenn die Anforderungen als angemessen bewertet werden und ausreichend persönliche Ressourcen verfügbar sind (Schiefele und Jacob-Ebbinghaus 2006). Aus empirischer Sicht besitzen Selbstberichte zur Wahrnehmung von Studienanforderungen, insbesondere zu Lernaktivitäten und Studienorganisation, prädiktive Validität für subjektive Studienleistungen (*r* = 0,44 bzw. *r* = 0,26) und -zufriedenheit (*r* = 0,32 bzw. *r* = 0,29; Jänsch und Bosse 2018).

## Die vorliegende Studie

Der pandemiebedingte Wandel im Hochschulbetrieb verändert die Anforderungen an das Studieren. Wir gehen von vier Annahmen aus. (i) Die technische Ausstattung sorgt als externe Ressource in prinzipieller Hinsicht dafür, dass Lernangebote überhaupt genutzt und das Studium organisiert werden können. (ii) Technikbereitschaft begünstigt als interne Ressource die Nutzung von Lernangeboten und die Organisation des Studiums, indem sie die aktive Annahme von Technik zur Bewältigung studienbezogener Aufgaben unterstützt. (iii) Beide Ressourcen sind mitbestimmend für die Nutzungsqualität und damit für die Zufriedenheit mit den digitalen Lernmedien, die während der Pandemie das Lernen und Studieren dominieren. Eine eingeschränkte Nutzungsqualität liegt bspw. dann vor, wenn eine Onlinelehrveranstaltung auf einem zu kleinen Anzeigegerät verfolgt werden muss oder ein Lernangebot wegen mangelnden Zutrauens in die eigenen Fähigkeiten nicht wie vorgesehen genutzt wird. (iv) Keine von beiden Ressourcen erscheint für sich genommen hinreichend. Erst das Zusammenspiel ermöglicht die Bewältigung eines rein technikvermittelten Studiums im Besonderen, aber auch technikgestützter Lernszenarien unter Normalbedingungen.

Den getroffenen Annahmen folgend geht die vorliegende Arbeit der Frage nach, in welchem Zusammenhang die beiden technikbezogenen Ressourcen mit der Bewältigung (1) von Lernaktivitäten, (2) der Studienorganisation und (3) der Zufriedenheit mit den Lernmedien in einem rein technikvermittelten (digitalen) Hochschulstudium stehen. Im Sinne eines hypothesenprüfenden Ansatzes wird ein positiver Einfluss beider Ressourcen auf die Bewältigung von Studienanforderungen und die Zufriedenheit mit digitalen Lernmedien unterstellt. Da wir von einer komplementären Nutzung beider Ressourcen im Studium ausgehen, wird außerdem eine Interaktionshypothese aufgestellt.

## Methode

Die vorliegende Untersuchung greift auf Daten der bereits oben zitierten Befragung von Studentinnen und Studenten im sächsischen Hochschulraum zurück (Karapanos et al. 2021). Der Datensatz basiert auf einer Onlinebefragung, die zwischen dem 30. April und 16. Juli 2020 durchgeführt wurde. Der Befragungszeitraum begann damit gut sechs Wochen nach den Campusschließungen und ungefähr drei (Universitäten) bzw. fünf Wochen (Fachhochschulen) nach dem Ende der vorlesungsfreien Zeit. Zur Rekrutierung von Teilnehmerinnen und Teilnehmern wurden die Studiendekaninnen und -dekane der staatlichen sächsischen Hochschulen angeschrieben und darum gebeten, die Einladung zur Befragung an die Studentinnen und Studenten der von ihnen verantworteten Studiengänge weiterzuleiten. Außerdem wurde über die Social-Media-Kanäle der Hochschulen zur Teilnahme aufgerufen. Nicht im Fokus der Befragung waren kleine Hochschulen mit weniger als 200 Studentinnen und Studenten, private Hochschulen, Stiftungshochschulen und die Hochschulen der sächsischen Polizei und Verwaltung.

### Stichprobe

**Abb. 1:**
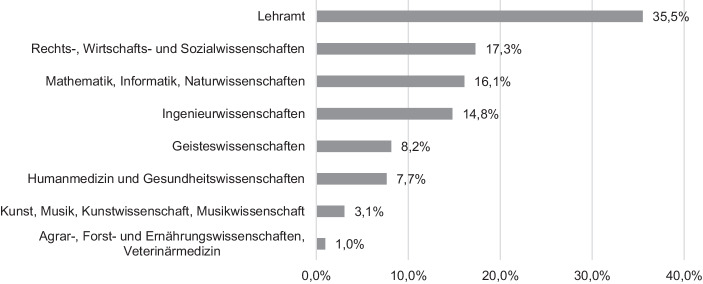
Stichprobenverteilung nach Studiendisziplin (Mehrfachnennung möglich)

Die Gesamtstichprobe des hier genutzten Datensatzes bestand aus 3469 Studentinnen und Studenten (32 % männlich, 64 % weiblich, 1 % divers), die im Sommersemester 2020 an staatlichen Hochschulen in Sachsen eingeschrieben waren. Mit Blick auf die Verteilung in der Grundgesamtheit nach Daten des statistischen Landesamts Sachsen (2020; Wintersemester 2019/20: 47,3 % weiblich) waren Frauen damit in der Stichprobe stark überrepräsentiert. Das galt auch für Studentinnen und Studenten der Universität Leipzig, der Heimatinstitution der Autoren. Stark unterrepräsentiert hingegen sind Studentinnen und Studenten der Technischen Universität Dresden, was wahrscheinlich auf konkurrierende lokale Befragungen zurückzuführen ist. Tab. 5 des Appendix stellt die Verteilung auf die einzelnen Hochschulen in der Stichprobe der Verteilung in der Grundgesamtheit gegenüber. Das mittlere Alter der Befragten lag bei 23,3 Jahren (*Mdn* = 22, *SD* = 5,13) und entsprach damit etwa dem Altersdurchschnitt in der Grundgesamtheit (*Mdn* = 23; Statistisches Landesamt Sachsen 2020).^2^ Abb. 1 zeigt die Verteilung der Befragten auf die Studiendisziplinen.

### Messinstrumente

Aus dem Datensatz werden 5 Items zur technischen Ausstattung sowie die 12 Items der Kurzskala zur Erfassung von Technikbereitschaft (Neyer et al. 2012) herangezogen. Als abhängige Variablen werden zwei Skalen aus dem Messinstrument für die Wahrnehmung von Studienanforderungen (Jänsch und Bosse 2018) und 15 Items zur Zufriedenheit mit digitalen Medien in Lehrveranstaltungen entnommen. Die Beantwortung der Items erfolgte mittels 5‑stufiger Skalen (Wertebereich −2 bis +2), wobei hohe Werte für eine hohe Merkmalsausprägung stehen.

#### Technische Ausstattung

Die Items zur technischen Ausstattung sind adhoc konstruiert. Sie orientieren sich an typischen Voraussetzungen für die Teilnahme an digitaler Lehre („*Ich habe eine stabile Internetverbindung.*“). Tab. 6 im Appendix zeigt die Items und berichtet die zugehörigen deskriptivstatistischen Kennwerte. Abb. 3 im Appendix bietet eine grafische Aufbereitung der Verteilungen. Alle Itemmittelwerte liegen deutlich über dem theoretischen Skalenmittelpunkt. Die Zustimmung zu diesen Items fiel also insgesamt hoch aus. Eine mit allen fünf Items durchgeführten Hauptachsenanalyse extrahierte genau einen Faktor mit einem Eigenwert > 1. Der Faktor besitzt den Eigenwert 2,21 und erklärt 44,3 % der Gesamtvarianz. Alle Items laden (≥ 0,46) auf diesen Faktor (vgl. Tab. 6 im Appendix). Aus den Items wird für die nachfolgende Analyse eine Skala zur Technikausstattung gebildet.

#### Technikbereitschaft

Technikbereitschaft wurde mithilfe der Kurzskala von Neyer et al. (2012) in den Facetten Technikakzeptanz („*Ich finde schnell Gefallen an technischen Neuentwicklungen.*“), Technikkompetenzüberzeugungen („*Im Umgang mit moderner Technik habe ich oft Angst zu versagen.*“) und Technikkontrollüberzeugungen („*Ob ich erfolgreich in der Anwendung moderner Technik bin, hängt im Wesentlichen von mir ab.*“) erfasst. Alle Items der Subskala zu Technikkompetenzüberzeugungen sind negativ formuliert. Zur einfacheren Interpretierbarkeit der Ergebnisse werden die Messwerte invertiert berichtet. Die Skala weist einen starken Deckeneffekt auf. Die Befragten schrieben sich also mehrheitlich ein hohes Maß an Technikkompetenz zu. Die Reliabilitäten sind sowohl auf Ebene der Subskalen als auch für die Gesamtskala als gut zu bewerten (vgl. Tab. 1).

**Tab. 1:** Bivariate Pearson-Korrelationen, deskriptivstatische Kennwerte und Reliabilitäten

		1	2	3	4	5	6	7	8
1	Technikausstattung	–							
2	Technikbereitschaft	0,31	–						
3	Technikakzeptanz	0,21	0,83	–					
4	Technikkompetenz	0,32	0,81	0,52	–				
5	Technikkontrolle	0,21	0,69	0,37	0,35	–			
6	Lernaktivitäten	0,27	0,20	0,15	0,17	0,16	–		
7	Studienorganisation	0,35	0,21	0,16	0,17	0,18	0,59	–	
8	Zufriedenheit mit digitalen Lernmedien	0,38	0,14	0,06	0,11	0,18	0,40	0,45	–
	Anzahl der Items	5	12	4	4	4	4	4	15
	*M*	1,18	0,51	0,09	0,87	0,53	−0,80	−0,38	0,56
	*SD*	0,78	0,75	1,04	1,01	0,80	0,93	0,79	0,76
	Cronbachs α	0,78	0,87	0,85	0,87	0,72	0,82	0,75	–
	McDonald’s ω	0,79	0,87	0,85	0,88	0,74	0,83	0,76	–

*Anmerkung. *2996 ≤ *N* ≤ 3332, *p* < 0,001 für alle Korrelationskoeffizienten, theoretische Spannweite der Skalen: −2 bis +2

#### Wahrnehmung von Studienanforderungen

Als zentrale Anforderungen im rein technikvermittelten Studium werden die Bewältigung von Lernaktivitäten und Studienorganisation betrachtet. Dazu wurde auf zwei Subskalen aus dem Messinstrument für die Wahrnehmung von Studienanforderungen (Jänsch und Bosse 2018) zurückgegriffen. Die Befragten sollten dazu beurteilen, wie viel leichter oder schwerer es ihnen im Vergleich zum Vorsemester fiel, mit Lernaktivitäten („*die Menge an Lernstoff zu bewältigen*“) und studienorganisatorischen Aufgaben („*passende Informations- und Beratungsangebote zu finden*“) umzugehen. Das Instrument wurde für die retrospektive Anwendung konstruiert und validiert (Jänsch und Bosse 2018). Die Reliabilität ist für beide Skalen als akzeptabel bzw. gut zu bewerten (vgl. Tab. 1).

**Abb. 2:**
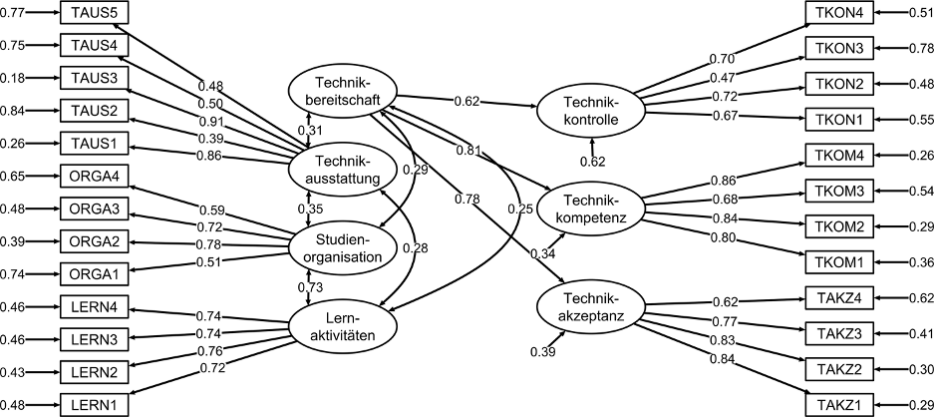
Messmodell mit standardisierten Parameterschätzungen

**Abb. 3:**
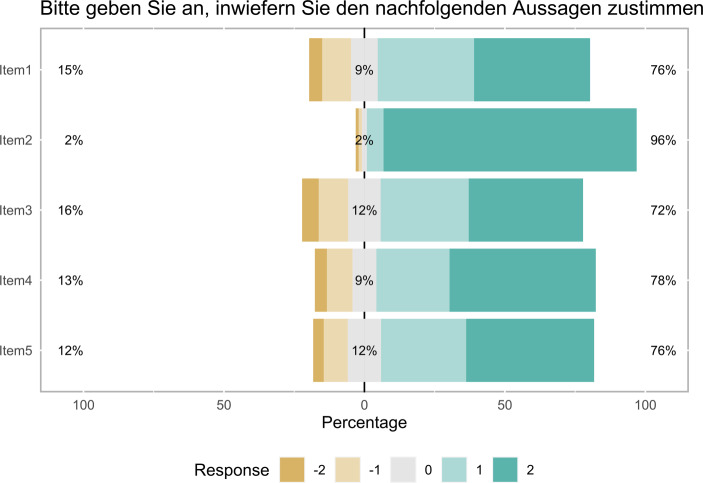
Instruktion: „Dozierende setzen gegenwärtig vermehrt digitale Medien und Anwendungen für die Lehre ein. Doch wie steht es um Ihre persönlichen Voraussetzungen zur Nutzung? Bitte geben Sie an, inwiefern Sie den nachfolgenden Aussagen zustimmen oder nicht zustimmen.“

Eine konfirmatorische Faktorenanalyse mit Full-Information-Maximum-Likelihood-Parameterschätzung (FIML) für die Skalen zur Wahrnehmung von Studienanforderungen, Technikausstattung und Technikbereitschaft (Second Order Factor, vgl. Abb. 2) weist auf eine gute Passung zwischen empirischer Datenstruktur und Messmodell hin (χ^2^ = 2262, *df* = 260, *p* < 0,001, CFI = 0,941, TLI = 0,932, SRMR = 0,050, RMSEA = 0,047).

#### Zufriedenheit mit den digitalen Lernmedien

Um zu erfassen, wie zufrieden die Befragten mit den digitalen Lernmedien im Sommersemester 2020 waren, wurden sie gebeten, ein Rating (*−2 sehr unzufrieden … +2 sehr zufrieden*) für 15 verschiedene Medientypen (z. B. digitale Texte, Auswahl in Anlehnung an Persike und Friedrich 2016, vgl. Tab. 7 im Appendix) vorzunehmen. Da meist nicht alle der aufgeführten Medientypen bei den Befragten im Studium zum Einsatz kamen, weisen die Daten in hohem Umfang fehlende Werte auf. Die Spannweite reicht von 14 % fehlende Werte für die häufig genutzten Chat‑/Konferenz-Dienste bis zu 85 % für die selten zum Einsatz gekommenen *Massive Open Online Courses*. Paarweise Korrelationsanalysen für die Zufriedenheitsratings der einzelnen Medientypen weisen ausnahmslos auf positive Zusammenhänge hin (0,16 ≤ *r* ≤ 0,79). Der Median aller Koeffizienten liegt bei *r* = 0,44. Daher erscheint eine Indexbildung als einfacher Mittelwert für das hier angedachte Vorhaben als gerechtfertigt und ausreichend.

### Analysestrategie

Für den Ergebnisbericht werden drei lineare Strukturgleichungsmodelle unter Anwendung des R‑Pakets *lavaan* (Rosseel 2012) berechnet. Neben Technikbereitschaft und -ausstattung werden auch Alter, Geschlecht, angestrebter Studienabschluss und die Fachdisziplin als Prädiktoren in die Analyse einbezogen. Technikbereitschaft wird wie bereits bei der Überprüfung des Messmodells als reflektives Konstrukt zweiter Ordnung aufgefasst. In der Literatur liegen zahlreiche Hinweise vor, dass sich Männer und Frauen bzw. Jungen und Mädchen in ihren technikbezogenen Selbstbewertungen unterscheiden können, wobei Männer und Jungen dazu neigen, sich zu überschätzen (Hatlevik et al. 2018; Ihme und Senkbeil 2017; Palczyńska und Rynko 2020). Trotz höherer technikbezogener Selbstwirksamkeitserwartungen von Männern und Jungen (Hedges *g* = 0,23 in Borokhovski et al. 2018; *g* = 0,18 in Cai et al. 2017) zeigen Frauen und Mädchen in standardisierten ICT-Tests im Mittel oft bessere Leistungen (*g* = 0,12 in Siddiq und Scherer 2019). Um diesem Umstand Rechnung zu tragen, wird neben dem Interaktionsterm Technikbereitschaft * Technikausstattung ein zweiter Interaktionsterm Technikbereitschaft * Geschlecht im Modell spezifiziert. Befragte, die sich der Geschlechterkategorie divers zuordneten (*n* = 29), werden aufgrund der geringen Fallzahl und weil nicht eindeutig bestimmt von der Analyse ausgeschlossen.

## Ergebnisse

**Abb. 4:**
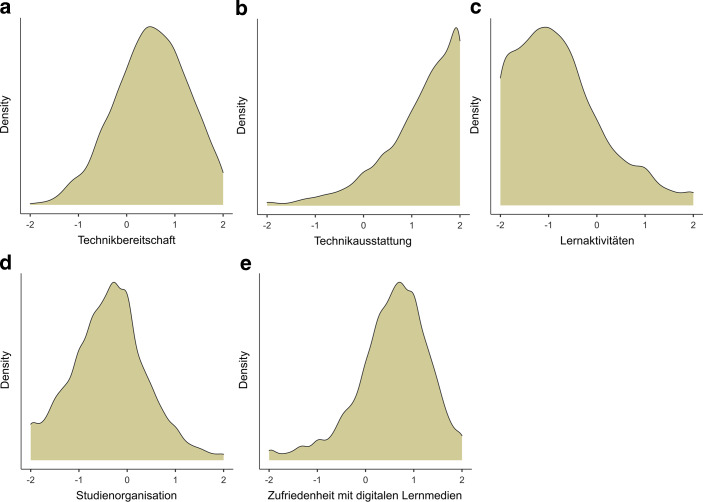
Dichteplots der Variablen Technikbereitschaft, Technikausstattung, Studienorganisation, Lernaktivitäten und Zufriedenheit mit digitalen Lernmedien

Für den Bericht der deskriptivstatistischen Kennwerte werden Skalenscores als Mittelwert gebildet, sodass alle Skalen unabhängig von der Anzahl zugehöriger Items einen Wertebereich von −2 bis +2 aufweisen. Fehlende Werte werden durch den individuellen Skalenmittelwert ersetzt, wenn für die befragte Person wenigstens 3 Items einer Skala beantwortet wurden. Die Mittelwerte, Standardabweichungen, Reliabilitätskoeffizienten und die Koeffizienten der Skaleninterkorrelationen können Tab. 1 entnommen werden. Abb. 4 im Appendix zeigt die zugehörigen Dichteplots.

Die weitere Analyse erfolgt mittels Strukturgleichungsmodellen in zwei Schritten. Im ersten Schritt (Nullmodell) gehen die Variablen Geschlecht, Fachdisziplin, Studienabschluss und Alter ein, im zweiten Technikbereitschaft und -ausstattung. Auf diese Weise kann sehr einfach anhand eines Vergleichs des Determinationskoeffizienten *R*^*2*^ die Erklärungskraft von Technikbereitschaft und -ausstattung für die jeweilige abhängige Variable bestimmt werden. Berichtet werden zugunsten einer kompakteren Darstellung jeweils nur die vollständigen Modelle. Die Angaben zu *R*^*2*^ beziehen sich nachfolgend immer auf das vollständige Modell, Δ*R*^*2*^ gibt dabei den Abstand zum Nullmodell an. Die Referenzkategorie für das Merkmal Geschlecht ist ‚weiblich‘. Da es prinzipiell möglich ist, mehrere Studiengänge parallel zu studieren, konnten sich die Befragten in mehreren Fachdisziplinen verorten (*n* = 179) und mehrere Abschlussarten als Studienziel angeben (*n* = 35). Um diese Personen – da uneindeutig – und solche mit fehlenden Werten in den Merkmalen Fachdisziplin und Studienabschluss nicht aus der Analyse ausschließen zu müssen, wird für beide Merkmale jeweils eine Pseudokategorie als Referenzkategorie gebildet, in die Befragte mit fehlenden Antworten oder Mehrfachantworten aufgenommen werden. Die Koeffizienten für diese beiden Merkmale geben damit den Abstand zu Befragten an, die sich keiner/mehreren Fachdisziplin(en) bzw. keiner/mehreren Abschlussart(en) zuordneten. Fehlende Werte in den latenten Merkmalen werden über FIML-Schätzung adressiert. Somit verbleiben von 3469 Fällen im Datensatz 3332 Fälle für die Analyse.

Technikbereitschaft und -ausstattung von Studentinnen und Studenten erklären auf substantiellem Niveau Unterschiede in der Bewältigung der Lernaktivitäten (Δ*R*^*2*^ = 0,11, vgl. Tab. 2), der Studienorganisation (Δ*R*^*2*^ = 0,16, vgl. Tab. 3) und der Zufriedenheit mit den digitalen Lernmedien (Δ*R*^*2*^ = 0,13, vgl. Tab. 4). Für die Bewältigung der Lernaktivitäten und der Studienorganisation erweisen sich beide Ressourcen als vergleichbar bedeutsam. Die Konfidenzintervalle der standardisierten Pfadkoeffizienten weisen nicht auf statistisch bedeutsame Unterschiede zwischen beiden Ressourcen, Technikbereitschaft und -ausstattung, hin. Lediglich für die Zufriedenheit mit den digitalen Lernmedien scheint die Technikausstattung (β = 0,29 [0,25, 0,32]) gegenüber der Technikbereitschaft (β = 0,12 [0,08, 0,17]) von größerer Bedeutung zu sein. Die Koeffizienten der Interaktionsterme sind ausnahmslos nicht signifikant von Null verschieden. Das deutet darauf hin, dass Technikausstattung und -bereitschaft nicht komplementär wirken und dass sich der Zusammenhang zwischen Technikbereitschaft und den hier untersuchten abhängigen Variablen als geschlechtsinvariant darstellt.

**Tab. 2:** Ergebnisse des Strukturgleichungsmodells: Lernaktivitäten

						95 % CI	
	Est	*SE*	*z*	*p*	β	Lower	Upper
*Alter*	0,01	0,004	1,40	0,163	0,03	−0,01	0,06
*Geschlecht*							
Männlich	−0,18	0,05	−3,67	< 0,001	−0,08	−0,12	−0,04
*Fachdisziplin*							
Ingenieurwissenschaften	0,02	0,11	0,22	0,830	0,01	−0,06	0,08
Rechts‑, Wirtschafts- und Sozialwissenschaften	−0,14	0,10	−1,38	0,168	−0,05	−0,11	0,02
Mathematik, Informatik, Naturwissenschaften	−0,02	0,10	−0,19	0,848	−0,01	−0,07	0,06
Lehramt	−0,02	0,11	−0,23	0,821	−0,01	−0,10	0,08
Geisteswissenschaften	−0,08	0,12	−0,69	0,491	−0,02	−0,07	0,03
Humanmedizin und Gesundheitswissenschaften	0,16	0,11	1,39	0,165	0,04	−0,02	0,09
Agrar‑, Forst- und Ernährungswissenschaften, Veterinärmedizin	−0,17	0,22	−0,77	0,442	−0,02	−0,05	0,02
Kunst, Musik, Kunstwissenschaft, Musikwissenschaft	0,24	0,17	1,39	0,165	0,03	−0,01	0,07
*Abschluss*							
Bachelor	−0,33	0,17	−1,94	0,053	−0,15	−0,30	0,002
Master	−0,13	0,18	−0,71	0,477	−0,04	−0,13	0,06
Diplom	−0,52	0,19	−2,79	0,005	−0,15	−0,25	−0,04
Staatsexamen	−0,47	0,18	−2,60	0,009	−0,22	−0,38	−0,05
*Technikausstattung (TA)*	0,24	0,02	1,02	< 0,001	0,22	0,18	0,27
*Technikbereitschaft (TB)*	0,20	0,03	7,43	< 0,001	0,18	0,14	0,23
TA * TB	0,02	0,03	0,62	0,534	0,01	−0,03	0,06
TB * männlich	−0,01	0,06	−0,14	0,886	0,00	−0,05	0,04
*R*^*2*^	0,13						

*Anmerkung. *χ^2^ = 3414, *df* = 503, *p* < 0,001, CFI = 0,901, TLI = 0,893, SRMR = 0,042, RMSEA = 0,057

**Tab. 3:** Ergebnisse des Strukturgleichungsmodells: Studienorganisation

						95 % CI	
	Est	*SE*	*z*	*p*	β	Lower	Upper
*Alter*	−0,01	0,00	−2,01	0,045	−0,04	−0,08	−0,001
*Geschlecht*							
Männlich	−0,22	0,05	−4,20	< 0,001	−0,09	−0,14	−0,05
*Fachdisziplin*							
Ingenieurwissenschaften	−0,27	0,11	−2,36	0,018	−0,08	−0,15	−0,01
Rechts‑, Wirtschafts- und Sozialwissenschaften	−0,29	0,11	−2,70	0,007	−0,09	−0,16	−0,03
Mathematik, Informatik, Naturwissenschaften	0,05	0,11	0,44	0,663	0,02	−0,05	0,08
Lehramt	−0,05	0,11	−0,47	0,637	−0,02	−0,12	0,07
Geisteswissenschaften	−0,23	0,13	−1,81	0,070	−0,05	−0,10	0,00
Humanmedizin und Gesundheitswissenschaften	−0,16	0,12	−1,29	0,197	−0,04	−0,09	0,02
Agrar‑, Forst- und Ernährungswissenschaften, Veterinärmedizin	−0,66	0,23	−2,82	0,005	−0,06	−0,10	−0,02
Kunst, Musik, Kunstwissenschaft, Musikwissenschaft	−0,40	0,18	−2,20	0,028	−0,05	−0,09	−0,01
*Abschluss*							
Bachelor	−0,30	0,18	−1,65	0,099	−0,13	−0,28	0,02
Master	−0,06	0,19	−0,33	0,743	−0,02	−0,12	0,08
Diplom	−0,35	0,20	−1,76	0,078	−0,09	−0,20	0,01
Staatsexamen	−0,42	0,19	−2,18	0,029	−0,19	−0,36	−0,02
*Technikausstattung (TA)*	0,31	0,03	11,96	< 0,001	0,28	0,24	0,32
*Technikbereitschaft (TB)*	0,24	0,03	8,46	< 0,001	0,22	0,17	0,27
TA * TB	−0,02	0,04	−0,46	0,647	−0,01	−0,06	0,04
TB * männlich	0,06	0,06	0,93	0,351	0,02	−0,03	0,07
*R*^*2*^	0,19						

*Anmerkung. *χ^2^ = 3447, *df* = 503, *p* < 0,001, CFI = 0,894, TLI = 0,885, SRMR = 0,042, RMSEA = 0,058

**Tab. 4:** Ergebnisse des Strukturgleichungsmodells: Zufriedenheit mit digitalen Lernmedien

						95 % CI	
	Est	*SE*	*z*	*p*	β	Lower	Upper
*Alter*	−0,01	0,002	−3,18	0,001	−0,05	−0,09	−0,02
*Geschlecht*							
Männlich	−0,29	0,03	−9,72	< .001	−0,19	−0,22	−0,15
*Fachdisziplin*							
Ingenieurwissenschaften	−0,10	0,07	−1,49	0,136	−0,05	−0,11	0,02
Rechts‑, Wirtschafts- und Sozialwissenschaften	−0,11	0,06	−1,77	0,076	−0,05	−0,11	0,01
Mathematik, Informatik, Naturwissenschaften	−0,03	0,06	−0,47	0,642	−0,01	−0,07	0,04
Lehramt	0,02	0,07	0,23	0,820	0,01	−0,07	0,09
Geisteswissenschaften	0,00	0,07	0,04	0,965	0,00	−0,04	0,05
Humanmedizin und Gesundheitswissenschaften	−0,02	0,07	−0,33	0,741	−0,01	−0,06	0,04
Agrar‑, Forst- und Ernährungswissenschaften, Veterinärmedizin	−0,25	0,14	−1,82	0,069	−0,03	−0,07	0,002
Kunst, Musik, Kunstwissenschaft, Musikwissenschaft	−0,27	0,11	−2,51	0,012	−0,05	−0,09	−0,01
*Abschluss*							
Bachelor	−0,02	0,11	−0,18	0,858	−0,01	−0,15	0,12
Master	0,15	0,11	1,34	0,18	0,06	−0,03	0,15
Diplom	−0,06	0,12	−0,51	0,61	−0,02	−0,12	0,07
Staatsexamen	−0,07	0,11	−0,59	0,558	−0,04	−0,19	0,10
*Technikausstattung (TA)*	0,21	0,02	14,64	< 0,001	0,29	0,25	0,32
*Technikbereitschaft (TB)*	0,09	0,02	5,68	< 0,001	0,12	0,08	0,17
TA * TB	−0,04	0,02	−1,66	0,096	−0,03	−0,07	0,01
TB * männlich	0,03	0,04	0,92	0,358	0,02	−0,02	0,06
*R*^*2*^	0,17						

*Anmerkung. *χ^2^ = 3295, *df* = 402, *p* < 0,001, CFI = 0,885, TLI = 0,874, SRMR = 0,046, RMSEA = 0,061

Demgegenüber zeigt sich als Nebenbefund, dass es männlichen Studenten marginal schwerer fiel, Lernaktivitäten (β = −0,08) und Studienorganisation (β = −0,09) zu bewältigen, wobei dieser Effekt nicht durch Unterschiede in der Wahl der Studiendisziplin vermittelt ist. Auch waren sie etwas weniger zufrieden mit den angebotenen Lernmedien (β = −0,19). Mit Blick auf die 95 % Konfidenzintervalle zeigen sich in keinem der drei Modelle Unterschiede zwischen Befragten verschiedener Fachdisziplinen und Abschlussarten.

## Diskussion und Limitationen

Das Sommersemester 2020 bedeutete für die Mehrheit der Studentinnen und Studenten zweifelsfrei eine große Umstellung. Unterschiede in der Ausstattung mit schnellem Internet und leistungsfähigen End- und Peripheriegeräten wirkten sich dabei auf das Lernen, die Studienorganisation und die Zufriedenheit mit den digitalen Lernmedien ebenso aus wie Unterschiede in technikbezogenen Einstellungen und Überzeugungen. Ein komplementäres Verhältnis von Technikausstattung und Technikbereitschaft zeigte sich im Rahmen der Untersuchung nicht. Vielmehr wirken beide Ressourcen vermutlich eher additiv. Eine stärker ausgeprägte Technikbereitschaft scheint damit Ausstattungsmängel in gewissem Rahmen kompensieren zu können und umgekehrt. Beide Faktoren erklären Unterschiede in der Bewältigung von Studienanforderungen und in der Zufriedenheit mit digitalen Lernmedien in allenfalls moderatem Umfang (Δ*R*^*2*^ ≤ 0,16), was vermutlich primär auf die weit überwiegend gute technische Ausstattung und insgesamt hohe Technikbereitschaft der Studentinnen und Studenten zurückzuführen ist. Wenig technikbereite und schlechter ausgestattete Studentinnen und Studenten waren demzufolge aber durch Campusschließungen und die Verlagerung von Lehrveranstaltungen in den digitalen Raum etwas stärker nachteilig betroffen. Da es sich bei beiden untersuchten technikbezogenen Ressourcen um – aus pädagogisch-psychologischer Sicht – eher distale Faktoren für Lern- und Studienverhalten handelt, erscheinen die gefundenen Effekte beachtenswert. Negative Auswirkungen auf den Studienerfolg in den betroffenen Jahrgängen könnten eine Folge sein. Allerdings reagierten die Hochschulen auf die Pandemie nicht nur bei der Durchführung der Lehre, sondern auch im Hinblick auf Prüfungsmodalitäten und führten Erleichterungen wie etwa die Wertung von Prüfungen als Freiversuch ein. Eine Befragung, die zwischen Juni und August 2020 im deutschen Hochschulraum durchgeführt wurde, konnte trotz der erschwerten Studienbedingungen (noch) keine gestiegene Studienabbruchintention feststellen (Marczuk et al. 2021). Studien aus Österreich berichten eine insgesamt vergleichbare Ausgangslage (Greimel-Fuhrmann et al. 2021), sodass die Befunde der vorliegenden Untersuchung mit Vorsicht auch auf die Situation in Österreich übertragbar sein dürften.

Die hier vorgelegten Befunde sind mit Zurückhaltung aufzunehmen, weil Daten aus Selbstselektionsstichproben nur eingeschränkt generalisierbar sind (Döring und Bortz 2016). Da es sich zudem um eine Onlinebefragung handelte, ist nicht auszuschließen, dass diese stärker technikbereite und besser ausgestattete Teilnehmerinnen und Teilnehmer rekrutierte. Gleichwohl waren, das zeigen die Daten (vgl. Kap. 4), auch in nennenswertem Umfang Befragte mit geringerer Technikbereitschaft und schlechter technischer Ausstattung im Datensatz enthalten, sodass eine erste Annäherung an die Fragestellung möglich war.

Mit Alter, Geschlecht, Fachdisziplin und Studienabschluss wurde in der Untersuchung nur auf einige wenige im Datensatz verfügbare Variablen kontrolliert. Fachdisziplin und Studienabschluss erwiesen sich in der Analyse zudem als wenig bedeutsam. Da die Befragten bei den Items zu Lernaktivitäten und Studienorganisation aber gebeten wurden, die Situation im Vergleich zum Vorsemester zu bewerten, sind ggf. bestehende Unterschiede zwischen Disziplinen und Abschlussarten in diesen Bewertungen möglicherweise bereits ‚eingepreist‘.

Unberücksichtigt blieb als möglichweise konfundierender Faktor der sozioökonomische Status des Elternhauses. Großzügigere Unterhaltszahlungen erleichtern die Beschaffung leistungsfähiger Technik und erlauben gleichzeitig die Einrichtung einer lernförderlichen Arbeitsumgebung. Insbesondere akademisch gebildete Eltern können ihren Kindern zudem mehr Orientierung und Unterstützung bei Problemen und Fragen zum Studium bieten als Nichtakademiker. Schon Befragungen aus der Zeit vor der Coronapandemie zeigten, dass Akademikerkinder gegenüber ihren Kommilitoninnen und Kommilitonen aus nicht-akademischen Elternhäusern eine höhere soziale und akademische Integration im Studium aufweisen und seltener das Studium als Überforderung erleben (Isleib 2019).

Weitere Limitationen der vorliegenden Untersuchung sind vor allem messtheoretischer Natur. Die technische Ausstattung wurde mit einem adhoc entwickelten Befragungsinstrument erfasst. Die Erfassung im Selbstbericht erwies sich zwar als praktikabel, könnte in künftigen Studien jedoch um Messalternativen erweitert werden, z. B. in Form einer browsergestützten Erfassung von Hardwareparametern wie dem Vorhandensein einer Webcam oder der Bezeichnung des Grafikprozessors (Mowery und Shacham 2012; Nakibly et al. 2015). Weiterhin ist die technische Ausstattung der Studentinnen und Studenten nicht als statische Größe zu verstehen. Vielmehr richtet sie sich vermutlich dynamisch nach privaten und studienbezogenen Anforderungen. Es ist nicht auszuschließen, dass sich Studentinnen und Studenten bereits im Verlauf des Sommersemesters neue technische Geräte anschafften – insbesondere, wenn das Studium zunehmend unter den Ausstattungsmängeln zu leiden begann. Denkbar ist auch der Rückzug in das Elternhaus, um eine ausreichend stabile Internetverbindung abseits der eigenen Wohngemeinschaft zu gewährleisten oder der Nachkauf von Webcams und anderer Peripheriegeräte, sobald diese wieder zu akzeptablen Preisen verfügbar waren. Solche Anpassungsstrategien und auch technikbezogene Lernprozesse reduzieren im Zeitverlauf das Ausmaß interindividueller Unterschiede in den hier untersuchten Ressourcen und sind mit einer Querschnittsuntersuchung nicht abbildbar.

Auch die Erfassung der Technikbereitschaft über ein Befragungsinstrument ist mit Limitationen verbunden. So zeigen insbesondere Selbsteinschätzungen technikbezogener Kompetenzen meist nur schwache bis moderate Zusammenhänge mit objektiven testbasierten Messungen (Christoph et al. 2015; Ihme und Senkbeil 2017; Vonkova und Hrabak 2015). Gleichwohl sind es gerade die subjektiven Überzeugungen und generalisierten Bewertungen, die handlungsleitend wirken (Ajzen 1991). Alle Messungen weisen zudem mehr oder weniger stark ausgeprägte Boden- bzw. Deckeneffekte auf (vgl. Abb. 4 im Appendix), was zu Varianzeinschränkungen und damit in der Tendenz zu einer Unterschätzung der wahren Effekte führt.

Die Ergebnisse erweitern den an Hochschulen geführten Diskurs über studentische Ressourcen im Hinblick auf Studierfähigkeit und Studienerfolg. Während im vergangenen Sommer eine Umstellung auf digitale Lernmedien blitzartig stattfinden musste, kann künftiges Lehren und Lernen – auch nach der Pandemie – gezielter auf vorhandene Ressourcen abgestimmt werden. Kurzfristig sei Dozentinnen und Dozenten angeraten, die technische Ausstattung ihrer Kursteilnehmerinnen und -teilnehmer zu erfragen oder tendenziell niedrigschwellige Angebote zu unterbreiten, die auch Lernende mit geringer Technikbereitschaft ansprechen. Perspektivisch dürften mit zunehmend besserer Ausstattung und Umgang damit interindividuelle Unterschiede in diesen Merkmalen an Relevanz für die Bewältigung des Studiums und für die Zufriedenheit mit digitalen Lernmedien verlieren.

## Appendix

**Tab. 5:** Stichprobe und Grundgesamtheit

	Befragungsdaten Sommersemester 2020		Statistisches Landesamt – Daten Wintersemester 2019/20	
Hochschule	Anzahl	In %	Anzahl	In %
Hochschule für Bildende Künste Dresden	18	0,5	515	0,5
Hochschule für Grafik und Buchkunst Leipzig	16	0,5	508	0,5
Hochschule für Musik Dresden	10	0,3	611	0,6
Hochschule für Musik und Theater Leipzig	39	1,1	1163	1,2
Hochschule für Technik und Wirtschaft Dresden	330	9,7	4578	4,6
Hochschule für Technik, Wirtschaft und Kultur Leipzig	152	4,5	6139	6,1
Hochschule Mittweida	350	10,3	6805	6,8
Hochschule Zittau/Görlitz	127	3,7	2818	2,8
Technische Universität Bergakademie Freiberg	148	4,3	3880	3,9
Technische Universität Chemnitz	138	4,0	9873	9,9
Technische Universität Dresden	167	4,9	30.123	30,1
Universität Leipzig	1678	49,2	29.399	29,4
Westsächsische Hochschule Zwickau	236	6,9	3501	3,5
Gesamt	3465	100,0	99.913	100,0

*Anmerkung.* Vier Befragte gaben an, an einer anderen Hochschule in Sachsen zu studieren.

**Tab. 6:** Items zur technischen Ausstattung

Item		*M*	*SD*	λ
1	Ich habe ein eigenes, internetfähiges Gerät zur freien Verfügung	1,83	0,61	0,78
2	Ich habe eine stabile Internetverbindung	0,97	1,16	0,46
3	Meine Internetverbindung ist ausreichend schnell, auch für datenintensive Anwendungen (z. B. Videotelefonie, Livestreaming, Downloads)	0,90	1,21	0,83
4	Ich verfüge über alle hardware-technischen Voraussetzungen (z. B. Endgerät mit ausreichend großem Display, Mikrofon, Webcam), um digitale Lernangebote wahrzunehmen	1,12	1,16	0,61
5	Ich verfüge über alle software-technischen Voraussetzungen (z. B. Präsentationstools, Tools zur Dokumentenerstellung), um digitale Lernangebote wahrzunehmen	1,05	1,12	0,57

*Anmerkung. *3354 ≤ *N* ≤ 3366

**Tab. 7:** Zufriedenheit mit digitalen Lernmedien

Lernmedien	*N*	*M*	*SD*
Audio (z. B. Podcasts)	2303	0,72	1,14
Blogs	840	−0,28	1,04
Chat‑/Konferenz-Dienste (z. B. Skype, Adobe Connect)	2903	0,56	1,15
Cloud-Dienste (z. B. Dropbox, Google Drive)	1299	0,22	1,18
Digitale Präsentationstools (z. B. Powerpoint, Prezi)	2771	0,74	1,07
Digitale Texte (z. B. PDFs)	3128	0,53	1,19
E‑Assessments (z. B. Online-Tests)	1634	0,44	1,20
EDV-Programme	778	−0,02	1,14
Foren	2171	0,10	1,16
Lernmanagement-Systeme (z. B. moodle, OPAL)	2984	0,93	1,03
Massive Open Online Courses (MOOCs)	506	−0,31	1,01
Soziale Netzwerke (z. B. facebook, Twitter)	603	−0,23	1,16
Webbasierte kollaborative Dokumentbearbeitung	938	0,12	1,17
Wikis	879	−0,15	1,09
Video	2609	0,88	1,08

*Anmerkung.* Bewertung auf fünfstufiger Skala von (−2) sehr unzufrieden bis (+2) sehr zufrieden

## Footnotes

1 Information and Communication Technology.

2 Das statistische Landesamt weist kein Durchschnittsalter aus. Aus den berichteten Alterstabellen lässt sich aber ein Medianalter von 23 Jahren berechnen.
